# Health-Related Quality of Life and Influencing Factors of Pediatric Medical Staff During the COVID-19 Outbreak

**DOI:** 10.3389/fpubh.2020.565849

**Published:** 2020-10-22

**Authors:** Feng Huang, Zhe Yang, Yue Wang, Wei Zhang, Yan Lin, Ling-chao Zeng, Xun Jiang, Lei Shang

**Affiliations:** ^1^Department of Health Statistics and Ministry of Education Key Lab of Hazard Assessment and Control in Special Operational Environment, Fourth Military Medical University, Xi'an, China; ^2^Airforce Medical Center, Fourth Military Medical University, Beijing, China; ^3^Department of Pediatric, Tangdu Hospital, Fourth Military Medical University, Xi'an, China

**Keywords:** COVID-19, health related quality of life, pediatrics medical staff, mental health, intervention measures

## Abstract

**Objective:** To evaluate the health-related quality of life (HRQoL) status and explore its associated factors in pediatric medical staff during the COVID-19 epidemic so as to provide fundamental evidence for clinicians and administrators to formulate targeted intervention measures to improve the HRQoL and mental health status in pediatric medical staff during this, and future pandemics.

**Methods:** A cross-sectional study was conducted to investigate the HRQoL of pediatric medical staff. Univariable and multivariable logistic regression were used to analyze the associated factors.

**Results:** A total of 2,997 participants were recruited. Females scored worse than males in terms of emotional functioning (OR = 1.6, 95% CI: 1.2–2.1) and cognitive functioning (OR = 1.4, 95% CI: 1.1–1.8). The respondents aged 30–39 and 40–49 years scored worse in nearly all domains of HRQoL compared health care professionals under 30 years old. Respondents with high education had lower scores in physical functioning (OR = 1.3, 95% CI: 1.0–1.7) and emotional functioning (OR = 1.5, 95% CI: 1.2–1.9). Compared with doctors, nurses had higher scores in all domains except for summary score and worry. The respondents whose working places had not set up pediatric fever clinics and isolated observation areas independently had lower scores in all domains except for worry. The respondents who had ever treated patients with COVID-19 had lower scores in all domains.

**Conclusion:** During the COVID-19 outbreak, the HRQoL of pediatric medical staff decreased. The factors associated with HRQoL can be used to develop intervention to improve HRQoL in pediatric medical staff.

## Introduction

A Public Health Emergency concerning the novel Coronavirus (COVID-19) was issued in Wuhan, China on 31 December 2019 (1). The virus quickly spread in other regions in China and the epidemic has broken out in other countries at the same time. Confirmed patients have been found in 94 countries outside of China and more than 100,000 people have been infected globally (80,859 in China) by 7 March 2020 (2, 3). Since the outbreak of the pandemic, significant numbers of medical staff have been regularly required to work long shifts. These medical staff not only undertake high-intensity work, but also face the risk of infection. According to published literature, the outbreak of COVID-19 has caused mental health problems among medical staff and the general public worldwide (4–7). To improve the mental health of residents in China during this crisis, the Chinese National Health Commission has released guidelines for local authorities to promote psychological crisis intervention for patients, medical personnel and the public during the COVID-19 outbreak (8).

COVID-19 is primarily transmitted *via* respiratory droplets and contact. Fever and respiratory symptoms are two of the most significant clinical manifestations (9). According to published studies, pediatric outpatients (73.11%) and hospitalized patients (33.09%) are most likely to suffer from respiratory disease as compared with other types of illnesses (10). While the pediatric medical staff were at high risk of infection, their workload was also increased tremendously due to the additional safety protocols to minimize COVID-19 transmission within the pediatric wards (11–14). Additionally, since parents were not allowed into the wards due to COVID-19 restrictions, pediatric medical staff often faced higher professional pressure on a daily basis as a result of close parental oversight and had to take on additional roles as parental figures to care for the needs of the young patients (15–17). These considerations together may have an impact on health-related quality of life (HRQoL) of pediatric medical staff (18).

Therefore, in this study we aim to evaluate the HRQoL and the unique influencing factors associated with the HRQoL of pediatric medical staff during the COVID-19 pandemic. Additionally, we wanted to examine if pediatric medical staff of different demographics and working conditions were differentially impacted by the increased demands of the pandemic. This study provides fundamental evidence for clinicians and administrators to formulate targeted intervention measures to improve the HRQoL and mental health status in pediatric medical staff during this and future pandemics.

## Materials and Methods

### Design and Participants

This study featured a cross-sectional design based on an online survey on Questionnaire Star between 13 and 17 February 2020 disseminated via WeChat, which is the most widely used social media platform in China as face-to-face interviews could not be carried out during the outbreak. Participants were encouraged to forward the questionnaire to other pediatric medical staff. A total of 2,997 pediatric medical staff from 29 provinces in China were recruited, and participants filled out the questionnaire anonymously, voluntarily and independently. This study was approved by the ethical board of the Fourth Military Medical University and all participants provided written informed consent.

### Instrument

Data was collected *via* a self-administered online questionnaire. The first Section was related to the participants' socio-demographic characteristics, including age, gender, occupation, education, major, professional titles, hospital grade, hospital type, province, and place of residence. The second Section was related to COVID-19 protection, including whether the pediatric fever clinic and isolation observation area are set up independently, whether they have ever treated COVID-19 or suspected COVID-19 patients, whether their family or colleagues have COVID-19 or suspected COVID-19, whether their family or colleagues have come into contact with COVID-19 patients or suspected patients, and whether they have worked in the clinical field of infectious diseases. The third Section was related to HRQoL. Since the purpose of this study is to analyze the individual's HRQoL, after discussion with 5 experts, four sub-scales, including physical functioning(6 items), emotional functioning(5 items), social functioning (4 items), and cognitive functioning(5 items) were selected from the PedsQL^TM^ Family Impact Module scale (19) and considered in the questionnaire. In addition, considering the worry and panic that may be caused by the pandemic, we have included 4 items to evaluate worry status through expert discussion, resulting in a HRQoL scale featuring 5 sub-scales.

Each item of the HRQoL scale has 5-Likert response options: 0 (never a problem), 1 (almost never a problem), 2 (sometimes a problem), 3 (often a problem), and 4 (almost always a problem). The item is then linearly converted to a score of 100 (0 = 100, 1 = 75, 2 = 50, 3 = 25, 4 = 0), and the score of each subscale is the sum score of its items divided by the number of items. Therefore, the higher the score, the better the HRQoL (i.e., less negative impact) (20).

The Cronbach's α coefficient and Split-Half Coefficient were used to assess the reliability of the third Section of the questionnaire. The Cronbach's α coefficient and Split-Half Coefficient of the HRQoL scale and all its subscales were all above 0.70.

### Statistical Analysis

Continuous variables were presented as Mean ± standard deviation (*mean*±*sd*). Categorical variables were presented as frequencies and percentages [n (%)]. One-way analysis of variance (ANOVA) or *t*-test were used to compare scores among groups defined by each characteristic. Multiple forward stepwise logistic regression analyses (Entry = 0.05, Removal = 0.1) were used to explore the factors associated with HRQoL. In a previous study reported by Lee et al. ~27% of health care workers reported psychiatric symptoms during the 2003 SARS-CoV outbreak in Singapore (21, 22). As such, the dependent variables were the summary of HRQoL values and all their domains were converted into dichotomous variables (≤*P*_25_ = 1, >*P*_25_ = 0) to better categorize participants for logistic regression analysis, according to its 25^th^ percentile of the score, where participants below the 25th percentile were the more severely impacted group The independent variables were the demographic characteristics and COVID-19 protection-related characteristics. Statistical significance was set at *p* < 0.05. SPSS 23.0 software package for Windows was used to carry out all analyses.

## Results

### Demographic Characteristics and HRQoL

A total of 2,970 respondents correctly filled and submitted the questionnaires out of a total of 2,997 respondents, and the effective rate of questionnaire collection was 99.1%. The respondents represented 29 provinces, among which Shaanxi province accounted for 43.3% of the responses. The vast majority of the respondents (88.8%) were women and most (43.0%) were aged 30–39 years. 52.4% of subjects were doctors. The mean score of the Summary HRQoL were 69.7 ± 15.9, and the mean scores of its five subscales were 58.9 ± 19.0 for worry, 70.5 ± 19.1 for physical functioning, 71.1 ± 20.2 for emotional functioning, 71.5 ± 19.5 for cognitive functioning and 75.5 ± 18 for social functioning. Further details of the participants' characteristics can be found in Table 1.

**Table 1:** HRQoL based on socio-demographic characteristics (mean ± *sd*).

	***N* (%)**	**Physical functioning**	**Emotional functioning**	**Social functioning**	**Cognitive functioning**	**Worry**	**Summary score**
**Gender**							
Male	334 (11.2)	69.2 ± 19.2	73.2 ± 20.3	72.5 ± 18.9	70.5 ± 19.8	59.9 ± 19.8	69.3 ± 16.7
Female	2,636 (88.8)	70.6 ± 19.1	70.9 ± 20.2	75.9 ± 17.8	71.6 ± 19.5	58.7 ± 18.9	69.8 ± 15.8
*P*-value		0.193	0.046	0.001	0.324	0.269	0.601
**Age (years)**							
<30	805 (27.1)	74.9 ± 18.2	75.4 ± 19.9	79.5 ± 17.3	77.1 ± 19.1	59.9 ± 20.1	73.7 ± 15.6
30–39	1,277 (43.0)	69.3 ± 19.1	70.4 ± 20.5	75.2 ± 17.9	71.1 ± 19.6	57.8 ± 18.9	69.0 ± 16.0
40–49	541 (18.2)	66.8 ± 18.8	68.2 ± 19.0	71.7 ± 18.2	65.4 ± 18.0	58.3 ± 18.2	66.2 ± 15.0
≥50	347 (11.7)	69.9 ± 19.7	68.5 ± 19.8	73.6 ± 17.7	69.5 ± 19.0	61.3 ± 17.5	68.7 ± 15.7
*P*-value		<0.001	<0.001	<0.001	<0.001	0.006	<0.001
**Education**							
Bachelor and below	2,540 (85.5)	71.2 ± 18.9	72.3 ± 20.1	76.4 ± 17.7	72.4 ± 19.4	59.5 ± 19.0	70.6 ± 15.7
Master and above	430 (14.5)	66.1 ± 19.3	64.5 ± 19.6	70.4 ± 18.7	66.5 ± 19.4	54.9 ± 18.3	64.7 ± 15.9
*P*-value		<0.001	<0.001	<0.001	<0.001	<0.001	<0.001
**Occupation**							
Doctors	1,557 (52.4)	67.7 ± 18.9	68.4 ± 19.8	72.1 ± 18.1	68.0 ± 19.1	58.6 ± 17.7	67.1 ± 15.4
Nurses	1,413 (47.6)	73.5 ± 18.9	74.2 ± 20.2	79.3 ± 17.1	75.4 ± 19.2	59.2 ± 20.3	72.6 ± 15.9
*P*-value		<0.001	<0.001	<0.001	<0.001	0.390	<0.001
**Professional titles**							
Senior	747 (25.2)	67.5 ± 19.3	68.3 ± 19.8	71.8 ± 17.9	67.1 ± 18.2	59.4 ± 17.6	67.0 ± 15.4
Intermediate	883 (29.7)	69.0 ± 19.3	69.3 ± 19.9	74.5 ± 18.1	69.4 ± 19.8	57.8 ± 19.2	68.2 ± 15.9
Junior	1,340 (45.1)	73.1 ± 18.5	73.9 ± 20.2	78.3 ± 17.5	75.3 ± 19.3	59.2 ± 19.5	72.3 ± 15.8
*P*-value		<0.001	<0.001	<0.001	<0.001	0.122	<0.001
**Hospital grade**							
Tertiary class-A hospital	2,172 (73.1)	70.6 ± 19.3	71.0 ± 20.5	75.9 ± 18.0	72.2 ± 19.5	58.2 ± 19.3	69.8 ± 16.1
Second class hospital	798 (26.9)	69.9 ± 18.6	71.4 ± 19.3	74.5 ± 18.0	69.8 ± 19.4	60.7 ± 18.1	69.4 ± 15.4
*P*-value		0.372	0.655	0.065	0.003	0.001	0.540
**Hospital type**							
Comprehensive hospital	2,224 (74.9)	70.6 ± 19.4	71.6 ± 20.1	75.7 ± 18.0	71.5 ± 19.6	59.4 ± 19.1	70.0 ± 16
Specialized hospital	746 (25.1)	70.0 ± 18.3	69.8 ± 20.4	75.0 ± 18.1	71.5 ± 19.3	57.1 ± 18.4	69 ± 15.7
*P*-value		0.475	0.033	0.354	0.956	0.004	0.124
**Pediatrics major**							
Internal medicine	1,836 (61.8)	70.4 ± 19.1	70.9 ± 19.9	74.9 ± 17.9	71.2 ± 19.4	59.2 ± 19.1	69.6 ± 15.8
Respiratory	193 (6.5)	71.0 ± 19.4	72.6 ± 22.5	76.1 ± 18.4	72.0 ± 18.6	58.1 ± 20.7	70.2 ± 16.8
Infection	79 (2.7)	71.6 ± 19.0	74.8 ± 20.8	77.0 ± 17.6	74.2 ± 21.4	60.4 ± 17.4	71.8 ± 15.7
Critical medicine	274 (9.2)	69.5 ± 20.0	71.6 ± 19.9	76.8 ± 17.7	73.6 ± 20.3	58.6 ± 18.8	70.2 ± 16.2
Others	588 (19.8)	70.6 ± 18.7	70.7 ± 20.2	76.5 ± 18.2	70.9 ± 19.5	58.0 ± 18.3	69.6 ± 15.6
*P*-value		0.879	0.375	0.196	0.214	0.615	0.726
**Place of residence**							
City	2730 (91.9)	70.5 ± 19.2	71.1 ± 20.2	75.5 ± 18.0	71.6 ± 19.4	58.9 ± 18.9	69.8 ± 15.9
Rural	240 (8.1)	70.1 ± 18.2	71.3 ± 19.7	75.6 ± 17.6	70.8 ± 20.2	58.5 ± 19.6	69.5 ± 15.7
*P*-value		0.788	0.932	0.955	0.561	0.739	0.793
**Province**							
Hubei	83 (2.8)	63.6 ± 15.9	56.8 ± 18.3	67 ± 16.9	63.8 ± 16.6	41.0 ± 15.0	59.0 ± 12.2
Others	2,887 (97.2)	70.7 ± 19.1	71.6 ± 20.1	75.8 ± 18	71.7 ± 19.5	59.4 ± 18.8	70.0 ± 15.9
*P*-value		0.001	<0.001	<0.001	<0.001	<0.001	<0.001
Total	2,970 (100.0)	70.5 ± 19.1	71.1 ± 20.2	75.5 ± 18	71.5 ± 19.5	58.9 ± 19.0	69.7 ± 15.9

Table 1 presents the univariate analyses results. Male respondents have higher scores than female respondents in emotional functioning but lower scores than female respondents in social functioning (72.5 vs. 75.9). The respondents under 30 years old had the highest scores in all HRQoL domains, while respondents aged 40–49 years old had the lowest scores. Respondents with higher education (Masters and above) had lower scores than those with lower education (Bachelors and below) in all domains. Along the same vein, doctors had lower scores in all domains except for worry when compared to nurses. Interestingly, respondents working in the tertiary class-A hospital had higher scores in social functioning and lower scores in worry compared to respondents working in second-class hospitals. Given that Hubei is the epicenter of the pandemic, respondents from the province had overall lower scores across all domains compared to respondents from other provinces.

### COVID-19 Protection Related Characteristics and HRQoL

As shown in Table 2, the hospitals in which 68.1% of the respondents worked had independent pediatric fever clinics and isolated observation areas. Notably, univariate analyses found that the respondents whose working places had not set up independent pediatric fever clinics and isolated observation areas had lower HRQoL scores, except for worry. The respondents who had treated patients with COVID-19 or suspected COVID-19 expectedly had lower scores than those who had not. The respondents whose family members or colleagues had ever suffered from COVID-19 or suspected COVID-19 had lower HRQoL than those who did not. The respondents whose family members or colleagues had ever contact with COVID-19 patients or suspected patients had lower scores in all domains. The respondents who had ever worked in the clinical field of infectious diseases had lower scores in all domains except for worry.

**Table 2:** HRQoL based on COVID-19 protection related characteristics ($\bar{x}\;\pm \;s$).

	***N* (%)**	**Physical functioning**	**Emotional functioning**	**Social functioning**	**Cognitive functioning**	**Worry**	**Summary score**
**The pediatric fever clinic and the isolated observation set up independently**							
No	946 (31.9)	68.1 ± 19.7	69.3 ± 20.1	73.0 ± 18.3	67.6 ± 19.5	58.0 ± 18.3	67.4 ± 16.0
Yes	2,024 (68.1)	71.6 ± 18.7	72.0 ± 20.1	76.7 ± 17.7	73.4 ± 19.2	59.3 ± 19.3	70.8 ± 15.7
*P*-value		<0.001	0.001	<0.001	<0.001	0.077	<0.001
**Whether you have ever treated patients with COVID-19 or suspected COVID-19**							
No	2,484 (83.6)	71.5 ± 19.1	72.2 ± 20.0	76.5 ± 17.8	72.4 ± 19.3	60.0 ± 18.9	70.7 ± 15.7
Yes	486 (16.4)	65.2 ± 18.4	65.7 ± 20.2	70.7 ± 18.4	67.0 ± 19.7	53.2 ± 18.5	64.6 ± 15.7
*P*-value		<0.001	<0.001	<0.001	<0.001	<0.001	<0.001
**Whether your family or colleagues have ever suffered from COVID-19 or suspected COVID-19**							
No	118 (4.0)	64.5 ± 18.2	64.4 ± 21.8	70.5 ± 18.2	66.7 ± 18.4	51.8 ± 18.8	63.8 ± 15.3
Yes	2,852 (96.0)	70.7 ± 19.1	71.4 ± 20.1	75.7 ± 17.9	71.7 ± 19.5	59.1 ± 18.9	70.0 ± 15.9
*P*-value		0.001	<0.001	0.002	0.006	<0.001	<0.001
**Whether your family or colleagues have ever contact with COVID-19 patients or suspected COVID-19**							
Yes	364 (12.3)	65.8 ± 17.2	65.3 ± 19.5	70.1 ± 17.8	67.0 ± 17.5	53.1 ± 17.6	64.6 ± 14.4
No	2606(87.7)	71.1 ± 19.2	72.0 ± 20.1	76.3 ± 17.9	72.2 ± 19.7	59.7 ± 19	70.5 ± 16.0
*P*-value		<0.001	<0.001	<0.001	<0.001	<0.001	<0.001
**Whether you have ever worked in the clinical field of infectious diseases**							
No	2,362 (79.5)	71.1 ± 19.0	71.8 ± 20.1	76.2 ± 17.9	72.3 ± 19.5	59.0 ± 19.1	70.3 ± 15.8
Yes	608 (20.5)	67.9 ± 19.3	68.7 ± 20.5	72.8 ± 18.2	68.4 ± 19.4	58.3 ± 18.5	67.4 ± 16.0
*P*-value		<0.001	0.001	<0.001	<0.001	0.381	<0.001
Total	2,970	70.5 ± 19.1	71.1 ± 20.2	75.5 ± 18.0	71.5 ± 19.5	58.9 ± 19.0	69.7 ± 15.9

### Factors Associated With HRQoL

As shown in Table 3, logistic regression analysis shows that females had lower emotional functioning scores (OR = 1.6, 95% CI: 1.2–2.1) and cognitive functioning scores (OR = 1.4, 95% CI: 1.1–1.8) when compared to males. In comparison to respondents below the age of 30, respondents aged 30–39 and 40–49 years had lower scores in all domains, except for worry. We also observed education level to be a factor that influenced HRQoL, where respondents with higher education level (Masters and above) had lower summary (OR = 1.5, 95% CI: 1.2–1.9), physical (OR = 1.3, 95% CI: 1.0–1.7) and emotional functioning (OR = 1.5, 95% CI: 1.2–1.9) scores compared to those with lower education level (bachelor and below). Nurses had higher scores in all domains, except for the summary score and worry, compared to doctors. Respondents living in Hubei Province had lower scores across all domains, except physical functioning, in comparison to those living in other Provinces. However, there was no statistical difference among respondents of different professional titles, hospital grade, hospital type, pediatrics major, and place of residence.

**Table 3:** Logistic regression analysis of HRQoL.

	**Physical functioning**	**Emotional functioning**	**Social functioning**	**Cognitive functioning**	**Worry**	**Summary score**
**Gender (ref** **=** **Male)**						
Female	–	1.6 (1.2–2.1)	–	1.4^*^ (1.1–1.8)		
**Age (ref** **=** **“ <30”)**						
30–39	1.6^**^ (1.3–2.0)	1.6^**^ (1.3–2.0)	1.3^*^ (1.1–1.7)	1.6^**^ (1.3–2.1)	1.1 (0.9–1.4)	1.8^**^ (1.4–2.2)
40–49	1.6^*^ (1.2–2.1)	1.7^**^ (1.3–2.2)	1.4^*^ (1.1–1.9)	2.0^**^ (1.6–2.8)	0.9 (0.8–1.2)	2.0^**^ (1.5–2.6)
≥50	1.3 (0.9–1.8)	1.3 (0.9–1.8)	1.1 (0.8–1.5)	1.5^*^ (1.1–2.1)	0.7^*^ (0.5–0.9)	1.3 (0.9–1.8)
**Education (ref** **=** **“Bachelor and below”)**						
Master and above	1.3^*^ (1.0–1.7)	1.5^*^ (1.2–1.9)	–	–		1.5^**^ (1.2–1.9)
**Occupation (ref** **=** **“Doctors”)**						
Nurses	0.8^*^ (0.6–0.9)	0.8^*^ (0.7–0.99)	0.6^**^ (0.5–0.7)	0.7^*^ (0.6–0.9)		
**Province (ref** **=** **“out of Hubei”)**						
Hubei	–	2.2^*^ (1.4–3.5)	1.8^*^ (1.1–2.9)	1.6^*^ (1.0–2.6)	6.3^**^ (3.4–11.5)	2.2^*^ (1.4–3.6)
**Whether the pediatric fever clinic and the isolated observation area are set up independently (ref** **=** **“Yes”)**						
NO	1.3^*^ (1.03–1.5)	1.2^*^ (1.01–1.4)	1.2^*^ (1.03–1.5)	1.6^*^ (1.3–1.9)		1.5^**^ (1.3–1.8)
**Whether you have ever treated patients with COVID–19 or suspected COVID–19 (ref** **=** **“No”)**						
Yes	1.3^**^ (1.1–1.7)	1.6^**^ (1.3–1.9)	1.5^*^ (1.2–1.8)	1.4^*^ (1.2–1.8)	1.6^**^ (1.3–2.0)	1.7^**^(1.4–2.1)
**Whether your family or colleagues have ever suffered from COVID-19 or suspected COVID-19 (ref** **=** **“No”)**						
Yes	1.8^*^ (1.2–2.6)	–	–	–	–	
**Whether your family or colleagues have ever contact with COVID-19 patients or suspected patients (ref** **=** **“No”)**						
Yes	–	–	1.3^*^ (1.0–1.6)	–	–	

* *indicates that the p value is less than 0.05*.

** *indicates that the p value is less than 0.001*.

Hospital environment was a factor that influenced HRQoL as well, where respondents whose workplace had no independent pediatric fever clinics and isolated observation areas had lower scores in all HRQoL domains than those who had, except for worry. As there is higher risk of infection, the respondents who had treated patients with COVID-19 or suspected COVID-19 had lower scores in all HRQoL domains than those had not treated patients with COVID-19 or suspected COVID-19. Similarly, the respondents whose family members or colleagues had ever suffered from COVID-19 or suspected COVID-19 had lower physical functioning scores (OR = 1.8, 95% CI: 1.2–2.6) than those whose family members or colleagues had not suffered from COVID-19 or suspected COVID-19. The same was found with social functional scores where those in contact with actual or suspected COVID-19 colleagues or family had lower scores (OR = 1.3, 95% CI: 1.0–1.6) than those did not. However, prior experience working in infectious disease departments has no effect on the outcome.

## Discussion

In a pandemic, health care workers face greater risk of infection and undertake higher work intensity as compared with the general population. This can lead to excessive fatigue and tension which led to anxiety, sadness, grievance, helplessness, and depression, among other emotions (23). A common thread across the different demographics and environmental situations is worry. Our results showed 8.2% of the respondents frequently felt anxious, and this is similar to the findings from Liu et al. (24). In addition, pediatric medical staff workers may face additional pressure as they have to take over the role of parents who were not able to freely visit and care for their children due to the restrictions placed by the pandemic. This could contribute to additional emotional and physical burden on these specific groups of medical staff (15–17).

Multivariate analysis showed that the socio-demographic characteristics associated with HRQoL of the respondents were gender, age, occupation and education. Females were associated with worse scoring than males in emotional functioning and cognitive functioning. Expectedly, the HRQoL of respondents living in Hubei Province was worse, which may be related to the more serious epidemic situation and higher risk of infection. We speculate that the HRQoL of doctors was worse than that of nurses because doctors receive patients first, and they need to conduct physical patient examinations (e.g., pharynx examinations), leading to a relatively higher risk of infection than nurse. In addition, doctors play vital roles in diagnosis and treatment planning. These roles require more effort in making decisions during treatment of patients and evaluating their recovery trajectory, experiencing more stress compared with nurses (25). Communication breakdowns between doctors and nurses working in neonatal wards have also been previously reported, and this could lead to considerable amount of emotional stress for doctors. A path to improve functioning of doctors within the wards could involve better communication between doctors and nurses, as well as reorganizing work schedules to allow more rest time between shifts (26). It is also important to foster trust and good relationships between the medical staff team within the wards (27).

Multivariate analysis also showed that HRQoL was closely related to COVID-19 protection-related characteristics, especially establishment of independent settings for the fever clinic and isolation area, as well as the treatment of patients with COVID-19 or suspected COVID-19. We hypothesize that these two factors were closely related to the risk of infection. The higher the possibility of infection, the more likely professionals are to suffer from anxiety (28). According to the joint investigation report from the China-World Health Organization and the relevant data released by the Chinese government, nosocomial infections among medical staff largely occurred in the early stage of COVID-19 infection, primarily in Wuhan when there was a lack of materials and experience in dealing with the disease (29). These findings suggest that it is critical to strengthen the safety of health care workers. Measures should be taken to reduce the risk of nosocomial infection, such as triage outside of hospitals (e.g., in tents or other shelters), establishment of an independent fever clinic and isolation area, and an adequate supply of protective equipment (30).

After the outbreak of the epidemic, the National Health Commission of China issued the guideline for emergency psychological crisis intervention during the outbreak of COVID-19 on January 26, 2020 (31). This guideline has formulated psychological intervention programs and key points for different personnel, such as people infected with COVID-19, personnel under quarantine, front-line staff, and the general public. According to our results, we believe that in addition to adopting the guidelines for daily psychological crisis intervention, we should also consider more targeted interventions according to the characteristics of pediatric medical staff to allay their concerns and improve their HRQoL. Pediatric medical staff who are 30–49 years old, of higher academic qualifications, doctors, and have a higher risk of infection should be given more attention. If the conditions permit, measures could be taken to meet their personal needs, such as care of an older family member and providing front-line staff with accommodations near the hospital. This would help maintain individual and team performance over the long run and improve the mental and physical health of these health care professionals. This is especially applicable for pediatric medical staff who have had contact with COVID-19 patients and suspected cases within their own family, as they face higher risks of infection as well as the additional emotional burden because of the health condition of their kin.

There are some limitations in this study. First, since this study is a cross-sectional survey it is not possible to elucidate causal relationships (7). Second, the survey was conducted online, which may result in respondent bias. However, face-to-face surveys were not possible during the pandemic. Finally, as we collected data from only medical staff working in pediatric wards, we are not able to generalize the findings of this study to other wards and medical staff workers.

## Conclusion

During the outbreak of COVID-19, the HRQoL of pediatric medical staff was impacted. The respondents with different demographic characteristics and COVID-19 protection-related characteristics were impacted to varying degrees. Therefore, clinicians and administrators should focus on developing interventions according to the characteristics of different groups to improve the HRQoL of medical staff.

## Data Availability Statement

The raw data supporting the conclusions of this article will be made available by the authors, without undue reservation.

## Ethics Statement

The studies involving human participants were reviewed and approved by ethical board of the Fourth Military Medical University. The patients/participants provided their written informed consent to participate in this study.

## Author Contributions

All authors on this manuscript made significant contributions to the study design. XJ and LS have made substantial contributions to design of the work. FH and ZY were responsible for the data analysis and interpretation of data, as well as drafting the manuscript. YW, WZ, YL, and L-cZ were involved in the acquisition of data. All authors read and approved the final manuscript.

## Conflict of Interest

The authors declare that the research was conducted in the absence of any commercial or financial relationships that could be construed as a potential conflict of interest.
